# A prospective study of lung disease in a cohort of early rheumatoid arthritis patients

**DOI:** 10.1038/s41598-020-72768-z

**Published:** 2020-09-24

**Authors:** A. Robles-Pérez, P. Luburich, S. Bolivar, J. Dorca, J. M. Nolla, M. Molina-Molina, J. Narváez

**Affiliations:** 1 ILD Unit, Department of Pneumology, Hospital Universitari de Bellvitge, Universitat de Barcelona, Feixa Llarga S/N, 08907 Barcelona, Spain; 2 Servei de Diagnòstic Per La Imatge El Prat (SDPI El Prat), Department of Radiology, Hospital Universitari de Bellvitge, Universitat de Barcelona, Barcelona, Spain; 3 Department of Rheumatology, Hospital Universitari de Bellvitge, Universitat de Barcelona, Barcelona, Spain

**Keywords:** Predictive markers, Prognostic markers, Rheumatoid arthritis, Respiratory tract diseases

## Abstract

Lung disease is common in patients with rheumatoid arthritis (RA). The onset of lung involvement in RA is not well known. The objective is to describe the features and evolution of lung involvement in early RA, its relationship with disease activity parameters, smoking and treatments. Consecutive patients with early RA without respiratory symptoms were included and tracked for 5 years. Lung assessment included clinical, radiological and pulmonary function tests at diagnosis and during follow-up. Peripheral blood parameters (erythrocyte sedimentation rate, C reactive protein, rheumatoid factor and anti-citrullinated peptide autoantibodies) and scales of articular involvement, such as DAS28-CRP, were evaluated. 40 patients were included and 32 completed the 5-year follow up. 13 patients presented lung involvement in the initial 5 years after RA diagnosis, 3 of them interstitial lung disease. Significant decrease of diffusion lung transfer capacity of carbon monoxide over time was observed in six patients, 2 of them developed interstitial lung disease. DLCO decrease was correlated with higher values of CRP and ESR at diagnosis. Methotrexate was not associated with DLCO deterioration or lung disease development. Subclinical progressive lung disease correlates with RA activity parameters. Smoking status and methotrexate were not associated with development or progression of lung disease.

## Introduction

Lung disease is commonly present in patients with rheumatoid arthritis (RA) and is a well-known cause of morbidity and mortality^1,2^. In some cases, lung disease is present at diagnosis of the rheumatic manifestations, occasionally preceding the joint symptoms^3,4^. However, the confirmation of lung disease is usually diagnosed after several months of presenting respiratory symptoms. The onset of lung impairment and its association with rheumatic disease or potential drug toxicity remains controversial since the majority of cases are identified months or years after RA diagnosis and treatment initiation. Therefore, it remains unknown if most interstitial lung disease (ILD)-RA cases present established fibrotic signs at diagnosis. Previous work has suggested an association between lung disease development and RA activity and inflammation^4–8^. Therefore, the identification of predictive factors of pulmonary involvement could help in optimizing lung screening for RA patients.

Treatments used for RA have been associated with both improvement and progression of lung disease. Data about the potential effect of methotrexate inducing pulmonary fibrosis are especially controversial, probably due to variability in methodology and difficulties in evaluating the lung state of these patients before initiating any RA treatment^9,10^. Furthermore, lung evaluation through pulmonary functional test including assessment of diffusing lung capacity for carbon monoxide (DLCO) and high-resolution computed tomography (HRCT) is not a common procedure in the initial evaluation of all RA patients. Therefore, to elucidate whether lung disease is induced by these treatments or associated to the RA itself remains a challenge.

The objectives of this work are: to describe the features of lung involvement during the first 5 years from RA diagnosis; to investigate the relationship between systemic disease activity parameters, treatments used for RA and smoking with the presence of lung abnormalities.

## Materials and methods

### Patient recruitment

In 2012, newly RA diagnosed patients at the outpatient clinic of the Rheumatology Department, University Hospital of Bellvitge, were included and followed-up for 5 years. The inclusion criteria were as follows: (1) age > 18 years; (2) diagnosis of RA according to the 1987 American College of Rheumatology classification criteria^11^; (3) duration of joint symptoms > 6 weeks and < 24 months; (4) previous treatment with nonsteroidal anti-inflammatory drugs, low dose of glucocorticosteroids (≤ 7.5 mg/day of prednisone or equivalent) or disease-modifying antirheumatic drugs (i.e., methotrexate or leflunomide) for no more than 3 months; and (5) absence of respiratory symptoms (patients were asked about dyspnea, using the modified Medical Research Council scale (mMRC), cough or wheezing). Subjects were excluded if they had other collagen vascular disorders, chronic respiratory or heart diseases prior to the RA diagnosis.

### Ethics approval

The study was conducted in accordance with the principles of the Declaration of Helsinki and the International Conference for Harmonization. Informed consent was obtained for every patient and the study was approved by the Clinical Research Ethics Committee of Bellvitge University Hospital.

### Clinical assessment

Clinical and epidemiological data were collected at RA diagnosis for inclusion. This included age, gender, duration of joint symptoms, smoking habits, the acute phase reactants erythrocyte sedimentation rate (ESR) and C-reactive protein (CRP), serological rheumatoid factor (RF) and anti-citrullinated peptyde autoantibodies (ACPA), swollen and tender joint count in 28 joints (DAS28-CRP score). ACPA levels (immunoglobulin G) were measured using a commercially available second-generation enzyme-linked immunosorbent assay kit: EliATM CCP Assay on an ImmunoCAP250 instrument (Phadia, Germany). During the same time, baseline respiratory assessment (dyspnea, cough, chest auscultation), chest radiograph (CR) and pulmonary function tests (PFT) were performed. The PFT included forced spirometry, for analyzing forced vital capacity (FVC), forced expiratory volume in the first second (FEV1), and pletismography for evaluating DLCO. All tests were done and interpreted at the Respiratory Department in accordance with the Spanish Society of Respiratory Medicine and Thoracic Surgery guidelines^11^.

During the follow-up, the same serological reactants and antibodies were collected and analyzed by a multidisciplinary team. The clinical, radiological and functional respiratory evaluation was repeated every year until the end of the study. In those patients with lung abnormalities in the CR, any abnormality in spirometry (defined as < 80% of FVC or FEV1 and/or DLCO), or significant decrease (> 10% on FVC and/or > 15% on DLCO) a high-resolution computed tomography (HRCT) of the chest was performed. The type of lung abnormality and the predominant radiological pattern on the HRCT were identified. HRCT features of ILD included ground glass opacities, septal thickening or reticulation, honeycombing, traction bronchiectasis and pulmonary nodules. The HRCT patterns were identified as usual interstitial pneumonia (UIP), including consistent UIP, probable UIP and indeterminate UIP, and non-UIP, including radiological features of non-specific interstitial pneumonia (NSIP) and other less frequently present patterns. Every HRCT was independently reviewed by two expert lung disease radiologists.

Information about treatment received for the rheumatologic disease and for the respiratory involvement (when applicable), was also collected during the follow up period.

### Statistical analysis

Categorical data are described as number of cases and percentage. Continuous variables are described as number of cases, mean, standard deviation (SD), median and interquartile range (Q1–Q3). The comparison between variables over time was performed with the t-test or Kruskal–Wallis test where applicable. For both FVC and DLCO a covariance analysis was performed to describe the evolution over time. This model was adjusted with other variables, such as ESR, CRP, ACPA, RF, DAS28-CRP, age, sex, smoking status and treatment for RA. Data were analyzed with R software 3.4.0 (https://cran.r-project.org/bin/windows/base/old/3.4.0/). *p* values of < 0.05 are considered statistically significant.

## Results

### Characteristics of the cohort

Forty patients (30 women) were recruited and followed-up. Mean age (SD) at inclusion was 47.1 (13.4) years. After 5 years, six patients had to discontinue the study because of a housing change, and two patients neglected to attend the last visits. Patient characteristics at inclusion and after 5 years are summarized in Table 1. Two patients presented ILD with preserved FVC at the initial lung evaluation: one NSIP and one organizing pneumonia (OP).

**Table 1:** Patient features at inclusion and after 5 years.

	2012	2017	*p*
Ever smoker, n (%)	15 (41.7)	19 (59.4)	–
FVC%	106 (15.8)	110 (19.7)	0.437
DLCO%	85.2 (20.3)	78.2 (17.5)	0.122
RF, IU/mL	25.6 (12.6–60.4)	66.0 (15.9–224)	0.201
ACPA, U/mL	116 (10.8–604)	115 (1–428)	0.710
CRP, mg/L	6.55 (2.92–15.7)	2.70 (0.75–6.47)	0.005
ESR, mm/h	26 (16–37.2)	19 (7.25–30)	0.080
DAS28-CRP	5.24 (4.48–6.05)	2.25 (1.96–3.38)	< 0.001

Results are expressed as mean (SD) or median (Q1–Q3) where applicable.

*FVC* forced vital capacity, *DLCO* diffusion lung transfer of carbon monoxide, *RF* rheumatoid factor, *ACPA* anti-citrullinated peptide autoantibodies, *CRP* C reactive protein, *ESR* erythrocyte sedimentation rate.

The values of *p* < 0.05 are considered statistically significant.

During the 5 years of follow up patients received different treatments for the rheumatologic condition: methotrexate, leflunomide, prednisone and biologic treatments such as infliximab, rituximab, certolizumab, abatacept and tocilizumab. The number of patients and duration of treatment is shown in Table 2. The most frequent treatment was methotrexate (71.9%). Six patients discontinued treatment with methotrexate. The reasons varied: improvement of joint involvement (3 patients), gastrointestinal adverse events (2 patients) and new-onset ILD (1 patient). One patient started methotrexate for his joint involvement. Six patients discontinued treatment with leflunomide. The reasons stated: worsening of joint pain that required another therapeutic strategy (5 patients) and headache (1 patient). Nine patients initiated glucocorticosteroids and five began a biologic medication. Changes on treatment over time were made by a rheumatologist according to disease evolution. Smoking habit increased over time (4 new cases after 5 years).

**Table 2:** Treatment used for rheumatoid arthritis in our cohort.

	2012, n (%)	2017, n (%)	Duration of treatment, months
Methotrexate	27 (67.5)	23 (71.9)	57.5 (7.1)
Leflunomide	10 (25)	7 (21.9)	50.7 (16.7)
Prednisone	4 (10)	13 (40)	50.8 (16.4)
Biologic treatment	0 (0)	5 (15.6)	24 (6–48)

Results are expressed as mean (SD) or median (Q1–Q3) were applicable.

Serological markers of RA activity ESR and CRP decreased after 5 years, while the mean value of RF increased. DAS28-CRP values were significantly lower after 5 years (Table 1). No significant correlation was observed between changes in these values and the different treatments used for the rheumatologic disease. No hospitalization was required, and no patient died during the 5 years of follow up.

### Features of pulmonary involvement

At the beginning of the study no patient presented respiratory symptoms, but ILD with preserved FVC was identified in two of them (5%) and diffuse cylindrical bronchiectasis in the absence of fibrosis in 7 of them (17.5%). In these cases the suspicion of lung disease was due to low DLCO values on PFT.

No significant differences in the mean PFT values, FVC or DLCO, at inclusion and after 5 years were observed in the overall cohort (Table 1). During the study, six patients (18.8%) showed a significant decrease of DLCO and underwent HRCT. Four of them (12.5%) presented radiologic abnormalities on the HRCT. Five of these patients (15.6%) presented mild exertional dyspnea (mMRC stage I). No other respiratory symptoms were observed.

Overall, the rate of lung involvement in our cohort at the first 5 years of RA diagnosis was 32.5%, from which 7.5% was ILD. Emphysema and diffuse cylindrical bronchiectasis in the absence of fibrosis were the most frequently identified abnormalities in the HRCT. One patient presented new onset of ILD after 5 years of RA diagnosis, with the HRCT pattern of UIP (see Supplementary Data).

The patient with NSIP at the beginning of the study discontinued follow-up and the patient with OP presented clinical and functional progression despite corticosteroid treatment. The seven patients presenting diffuse cylindrical bronchiectasis at baseline did not show radiologic progression or associated respiratory symptoms, such as increased volume of sputum or respiratory infections over 5 years. The main radiologic findings are summarized in Table 3.

**Table 3:** Radiologic findings in patients who underwent HRCT scan at follow up.

Patients with DLCO decrease	HRCT findings
Patient 1	Emphysema, cylindrical bronchiectasis
Patient 2	Emphysema, usual interstitial pneumonia
Patient 3	No radiological findings
Patient 4	Cylindrical bronchiectasis
Patient 5	Emphysema, cylindrical bronchiectasis, organizing pneumonia
Patient 6	No radiological abnormalities

*HRCT* high resolution computed tomography.

### Pulmonary involvement and risk factors

Smoking history was only present in 3 patients with pulmonary involvement and did not associate a worsening in pulmonary function parameters. Baseline values of ESR and CRP and change of ACPA value over time showed an inverse significant correlation with DLCO decrease. No correlation between FVC change and other variables was observed. The results were adjusted for smoking status, baseline FVC and DLCO values. Methotrexate was not associated with ILD development or progression. Contrary, a direct correlation between DLCO stability and methotrexate treatment was observed, even after adjusting for RA activity biomarkers. FVC and DLCO correlations are shown in Tables 4 and 5 respectively.

**Table 4:** Correlation between FVC evolution and different variables.

	B	CI	*p*
Age	− 0.20	− 057 to 0.16	0.253
Gender (male)	0.05	− 9.42 to 9.51	0.992
Ever smoker	5.84	− 0.01 to 0.04	0.193
RF	0.02	− 0.02 to 0.03	0.709
ACPA	0.00	0.00 to 0.01	0.816
CRP	0.06	− 0.10 to 0.22	0.418
ESR	0.23	− 0.02 to 0.48	0.067
DAS28-CRP	2.88	− 1.35 to 7.11	0.166
Methotrexate	− 1.87	− 11.70 to 7.97	0.690
Leflunomide	1.87	− 7.97 to 11.70	0.690
Prednisone	6.76	− 5.07 to 18.60	0.241

The values of *p* < 0.05 are considered statistically significant.

*RF* rheumatoid factor, *ACPA* anti-citrullinated peptide autoantibodies, *CRP* C reactive protein, *ESR* erythrocyte sedimentation rate.

**Table 5:** Correlation between DLCO evolution and different variables.

	B	CI	*p*
Age	− 0.04	− 0.04 to 0.33	0.842
Gender (male)	3.31	− 6.81 to 13.44	0.509
Ever smoker	− 2.15	− 11.20 to 6.89	0.630
RF	0.01	− 0.02 to 0.03	0.709
ACPA at diagnosis	0.00	− 0.01 to 0.01	0.883
ACPA evolution	− 0.01	− 0.01 to 0.00	0.005
CRP	− 0.27	− 0.45 to − 0.10	0.003
ESR	− 0.28	− 0.54 to − 0.03	0.028
DAS28-CRP	− 3.20	− 8.16 to 1.77	0.199
Methotrexate	8.95	0.10 to 17.79	0.048
Leflunomide	− 7.69	− 16.92 to 1.55	0.099
Prednisone	− 2.36	− 17.17 to 12.45	0.747

The values of *p* < 0.05 are considered statistically significant.

*RF* rheumatoid factor, *ACPA* anti-citrullinated peptide autoantibodies, *CRP* C reactive protein, *ESR* erythrocyte sedimentation rate.

## Discussion

In this single-center prospective cohort study of patients with early RA, we demonstrate that the systematic lung evaluation, including HRCT and DLCO, allows the identification of respiratory abnormalities in more than one-third of cases, most of them without symptoms. On the other hand, disease activity measured through scales and serum acute phase markers decreased after 5 years of follow up in most cases. This can be attributed to a better control of the inflammatory process associated to the treatment, as previously reported^13^.

No changes in mean lung function in the overall sample were observed. However, 6 patients (18.8%) presented a significant decrease in DLCO values, four of them with airway or parenchymal abnormalities on the HRCT. The variability in the reported prevalence of RA lung involvement could be due to the different protocols used^4,5,7,24–26^. If simple spirometry is used, these patients cannot be identified. In our work those patients with lung disease presented higher rates of inflammatory serological markers, such as ESR and CRP, in line with previous studies^14–17^. In our study, higher values of CRP and ESR at RA diagnosis associated a DLCO decrease over time. The presence of inflammatory proteins in the lungs has been associated with progressive lung disease^16^ and higher rates of ESR associate ground glass opacities on lung HRCT^15^. Sparks et al. reported a higher risk for the development of RA-ILD in patients with higher values of DAS28 in a prospective cohort of RA patients^8^. This association between local and systemic inflammation with lung disease suggested that a better control of the systemic rheumatoid process could associate a lower risk of developing progressive lung involvement. However, our data show that some RA patients present lung disorders over time despite an improvement in join symptoms or inflammatory markers. So, other factors associated with the RA could contribute to the lung involvement.

Furthermore, patients that present ILD several years after onset of RA and receive methotrexate are difficult to distinguish as RA-ILD or ILD induced by the drug because they usually lack an initial lung evaluation. The systematic lung functional evaluation from RA diagnosis showed that DLCO did not decrease in those patients treated with methotrexate. Only one patient stopped methotrexate because of lung involvement, but no clear toxicity was proven. Traditionally methotrexate had been related with lung toxicity, including new onset or worsening ILD, with toxicity rates up to 8%^18^. However, recent data advocate its safe use. Several meta-analysis of clinical trials have shown that treatment with methotrexate does not increase the risk of non-infectious lung disease as compared with other treatments for RA^19–21^. Furthermore, methotrexate use in RA is associated with survival^22,23^. The use of methotrexate in our study associated a good control of the rheumatic disease without higher risk for ILD. Nevertheless, performing HRCT scan and monitoring of lung function, especially in the first months of treatment would be recommended, as well as in other treatments for RA.

Our results suggest that lung disease may be identified at diagnosis and in the first years after RA diagnosis. Airway involvement is not uncommon in RA patients, especially in the form of bronchiectasis and/or bronchiolitis^7^. In our cohort these patients presented clinical and functional stability after 5 years and did not associate an increased risk of mortality, which differs from previous data^27,28^. However, respiratory infections could be more frequent in these cases after years and in the context of some biological treatments. Up to 6.3% of our cohort developed clinically significant ILD. Though radiological findings in the first years of RA are variable and non-specific as described in previous studies^4,5,7,24–26^, ILD can develop in early RA^3^. Clinically significant RA-ILD has been observed on average in 8 to 10% of patients with RA^3,29–31^. Therefore, ILD may develop at any time of the rheumatologic disease and, when no systematic evaluation is performed from RA diagnosis, ILD can not be certainly attributed to the disease or the treatment. Finally, an optimized management and early treatment of RA-ILD could change this less than optimal progression.

The main limiting factor of our study is the total number of patients included, similar to previous works that include early RA patients, since recruiting patients with recent RA diagnosis before receiving methotrexate or other immunomodulators for improving join pain or activity limitation remain difficult. Future multi-center studies should be performed to validate our results. Despite this, the present study helps to clarify the natural history of the disease by showing that lung involvement in RA is present even from the beginning of the rheumatic process and could be related to the disease activity.

In conclusion, a systematic clinical and PFT evaluation of the lung (including DLCO) enables early identification of RA-ILD and other types of lung involvement through HRCT. Patients at higher risk may be identified through serological markers of disease activity. Therefore, it could help optimize RA treatments and lung management from initial stages, in the attempt to avert progression to advanced stages. Finally, the use of methotrexate has not been associated with a higher incidence of lung involvement.

## Supplementary information

Supplementary Information.

## Data Availability

The datasets generated during and/or analysed during the current study are available from the corresponding author on reasonable request.
