# Supplementary material for: A prospective study of lung disease in a cohort of early rheumatoid arthritis patients
Source: Sci Rep. 2020 Sep 24;10:15640. doi: 10.1038/s41598-020-72768-z (PMC7515904; doi:10.1038/s41598-020-72768-z)
Supplement: Supplementary file 1 — Supplementary Information. [file 41598_2020_72768_MOESM1_ESM.docx]

**A prospective study of lung disease in a cohort of early rheumatoid arthritis patients**

Robles-Pérez A^1^, Luburich P^2^, Bolivar S^2^, Dorca J^1^, Nolla JM^3^, Molina-Molina M^1*^, Narváez J^3*^

^1^ ILD Unit, Department of Pneumology, Hospital Universitari de Bellvitge, Universitat de Barcelona, Barcelona, Spain.

^2^ Servei de Diagnòstic per la Imatge El Prat (SDPI El Prat), Department of Radiology, Hospital Universitari de Bellvitge, Universitat de Barcelona, Barcelona, Spain.

^3^ Department of Rheumatology, Hospital Universitari de Bellvitge, Universitat de Barcelona, Barcelona, Spain.

*Both authors equally contributed as senior author

**Corresponding author:**

Maria Molina-Molina

Pneumology Department, Hospital Universitari de Bellvitge

Feixa Llarga s/n 08907, Barcelona (Spain)

Phone number: +34 932607500 –ext 7689

[mariamolinamolina@hotmail.com](mailto:mariamolinamolina@hotmail.com)

**Case example**

Here we present a 56 years-old male patient who developed associated pulmonary fibrosis in the first years of the rheumatic disease.

RA was diagnosed after 18 months of joint symptoms. He had no tobacco history or other relevant exposures. Serological markers of disease activity were high, specially the anti-citrullinated auto-antibodies (ACPA = 606 IU/mL). PFT and chest radiograph at inclusion were normal and no respiratory symptoms were reported. The patient initiated low-dose prednisone and methotrexate. At year 4^th^ of follow up a decrease of >15% in DLCO without FVC impairment was observed and the patient reported mMRC stage I dyspnea. At this point serological markers of disease activity had increased (RF 90 IU/mL, ACPA 1180 IU/mL, DAS28-CRP 1.85). HRCT was performed and a consistent UIP pattern was found, although there were only few areas with the typical fibrotic changes (Figure 1). The case was evaluated by a multidisciplinary team with a rheumatologist, pneumologist and radiologist. Even though no clear toxicity was proven, methotrexate was stopped, and hydroxychloroquine and rituximab were associated to prednisone for better managing disease activity. After one year of treatment PFT values showed stability and the serological markers of disease activity improved (RF 52 IU/mL, ACPA 450 IU/mL).

The systematical lung assessment in this patient follow-up was useful for optimizing RA activity even when joint pain was under control. This case shows that UIP pattern might develop even in the first 5 years of the disease.


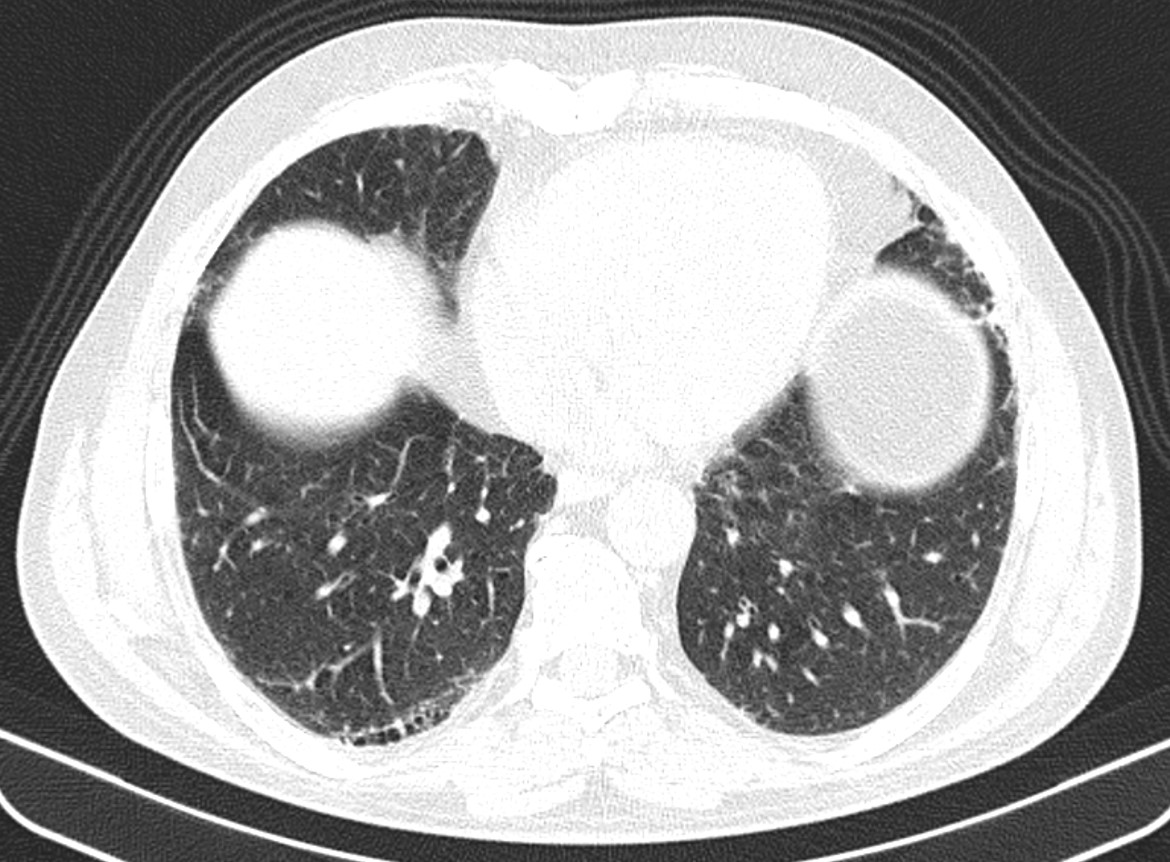


Figure 1. HRCT image showing septal thickening and subpleural honeycombing. HRCT: high resolution computed tomography.
